# Graphene oxide-based SPR biosensor chip for immunoassay applications

**DOI:** 10.1186/1556-276X-9-445

**Published:** 2014-08-28

**Authors:** Nan-Fu Chiu, Teng-Yi Huang, Hsin-Chih Lai, Kou-Chen Liu

**Affiliations:** 1 Institute of Electro-Optical Science and Technology, National Taiwan Normal University, No. 88, Sec. 4, Ting-Chou Road, Taipei 11677, Taiwan; 2 Department of Medical Biotechnology and Laboratory Science, Chang Gung University, No. 259 Wenhwa 1st Road, Gueishan Shiang, Taoyuan 33302, Taiwan; 3 Department of Electronic Engineering, Chang Gung University, No. 259 Wenhwa 1st Road, Gueishan Shiang, Taoyuan 33302, Taiwan

**Keywords:** Graphene oxide sheet (GOS), Surface plasmon resonance (SPR), Protein, Bovine albumin serum (BSA)

## Abstract

This work develops a highly sensitive immunoassay sensor for use in graphene oxide sheet (GOS)-based surface plasmon resonance (SPR) chips. This sensing film, which is formed by chemically modifying a GOS surface, has covalent bonds that strongly interact with the bovine serum albumin (BSA), explaining why it has a higher sensitivity. This GOS film-based SPR chip has a BSA concentration detection limit that is 100 times higher than that of the conventional Au-film-based sensor. The affinity constants (*K*_A_) on the GOS film-based SPR chip and the conventional SPR chip for 100 μg/ml BSA are 80.82 × 10^6^ M^-1^ and 15.67 × 10^6^ M^-1^, respectively. Therefore, the affinity constant of the GOS film-based SPR chip is 5.2 times higher than that of the conventional chip. With respect to the protein-protein interaction, the SPR sensor capability to detect angle changes at a low concentration anti-BSA of 75.75 nM on the GOS film-based SPR chip and the conventional SPR chip is 36.1867 and 26.1759 mdeg, respectively. At a high concentration, anti-BSA of 378.78 nM on the GOS film-based SPR chip and the conventional SPR chip reveals two times increases in the SPR angle shift. Above results demonstrate that the GOS film is promising for highly sensitive clinical diagnostic applications.

## Background

As a novel class of two-dimensional carbon nanostructures, graphene oxide sheets (GOSs) have received considerable attention in recent years in the fields of plasmonics [1-3] and surface plasmon resonance (SPR) biosensors [4-11], following both experimental and theoretical scientific discoveries. GOSs have remarkable optical [12-19] and biosensing [20-28] properties and are expected to have a wide range of applications. A GOS has a high surface area and sp^2^ within an sp^3^ matrix that can confine *π*-electrons [12-14,29]. GOSs contain oxygen at their surfaces in the form of epoxy (-O), hydroxyl (-OH), carboxyl (-COOH), and ether functional groups on a carbon framework [30-35]. The direct bandgap behavior of a GOS can be controlled by modification by oxidation that generates photoluminescence (PL) [16-19] and makes available chemically functionalized GOS with biological applications. Surface chemical modifications significantly influence the performance of surface chemistry-derived devices such as optoelectronic devices, luminescent devices, biosensors, and biomaterials. This work develops a novel method for detecting immunological diseases, in which terminal groups (-COOH) are modified and carboxyl groups on GOS surfaces are activated. The carboxyl groups of a GOS film can be converted into amine-reactive groups to increase its surface area sensing. Furthermore, modifying the oxygen-containing functional groups on the surface of GOS can increase its bandgap and its dielectric constant, thereby improving its surface plasmon resonance (SPR) properties.

## Methods

**Figure 1:**
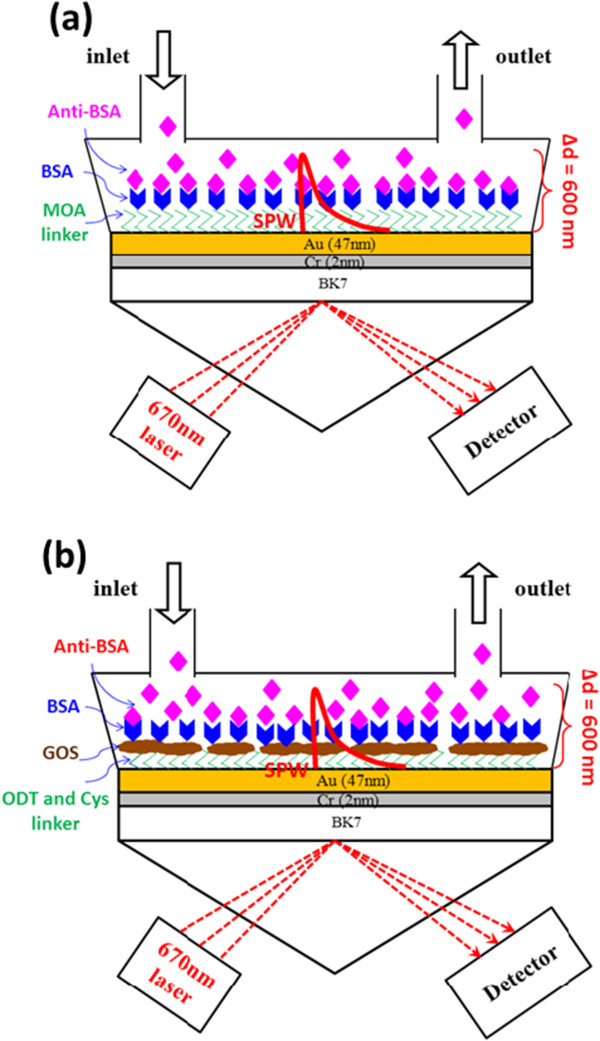
**SPR biosensor chip using an immunoassay method for detecting a protein using a gold binding. (a)** Conventional SPR chip and **(b)** GOS film-based SPR chip**.** Intensity of an evanescent field with a depth of approximately 100 ~ 500 nm decays exponentially with increasing distance from the metal. Bimolecular binding, observed within approximately 10 nm of the metal surface, gives rise to a higher signal shift response than that of the interactive process at a distance of 300 nm therefrom. For typical SPR Kretschmann prism coupling that uses a red light to induce the evanescent field, its field intensity is no more than 600 nm in practice.

Figure 1a,b shows the design of two sensing chips, i.e., a conventional SPR chip and a GOS film-based SPR chip. Standard SPR thin films were deposited with thin film for gold (Au) thickness of 47 nm and chromium (Cr) thickness of 2 nm on BK7 glass substrate to a thickness of 0.17 mm. SPR experiments were conducted using a BI-3000G SPR system with Kretschmann prism coupling (Biosensing Instrument, Tempe, AZ, USA). The test injection sample volume was 200 μl and the flow rate was 60 μl/s. All experiments were performed at 25°C and repeated in triplicate.

### Designed configuration for sensing

Figure 1a presents a conventional SPR sensing chip and a biomolecule binding mechanism. 8-Mercaptooctanoic acid (MOA; Sigma-Aldrich Co. LLC., St. Louis, MO, USA) is activation of carboxylic acid-terminated thiol self-assembled monolayers (SAMs) on a modified Au surface. MOA binds to the Au surface through their thiol linker (-SH end) resulting monolayers, which are terminated with carboxylic acid (-COOH). The MOA can be further functionalized to immobilize a bovine serum albumin (BSA; Sigma, Chemical Company, St. Louis, MO, USA) protein. Anti-BSA protein interactions are performed as well.

Figure 1b shows a GOS film-based SPR chip with its biomolecule binding mechanism. Two binding mechanisms are functionalized SAMs on amino-modified Au surfaces by solutions of cystamine (Cys; Alfa Aesar Co., Ward Hill, MA, USA) with a concentration of 5 mM and octadecanthiols (ODT, C_18_H_37_SH; Sigma-Aldrich Co. LLC.) with a concentration of 10 mM formation of Au-S bonds that immobilize a GOS. Concentration of the GOS diluted in water was 2 mg/ml. The GOS film sensing surface detects BSA protein concentrations in a range of 100 pg/ml to 100 μg/ml and their interaction with anti-BSA. Moreover, analysis is performed of the kinetics of protein-protein interactions at physical contacts that are established between two proteins, owing to biochemical events, protein affinity adsorption forces, and protein binding forces.

### Preparation of modified GOS films

**Figure 2:**
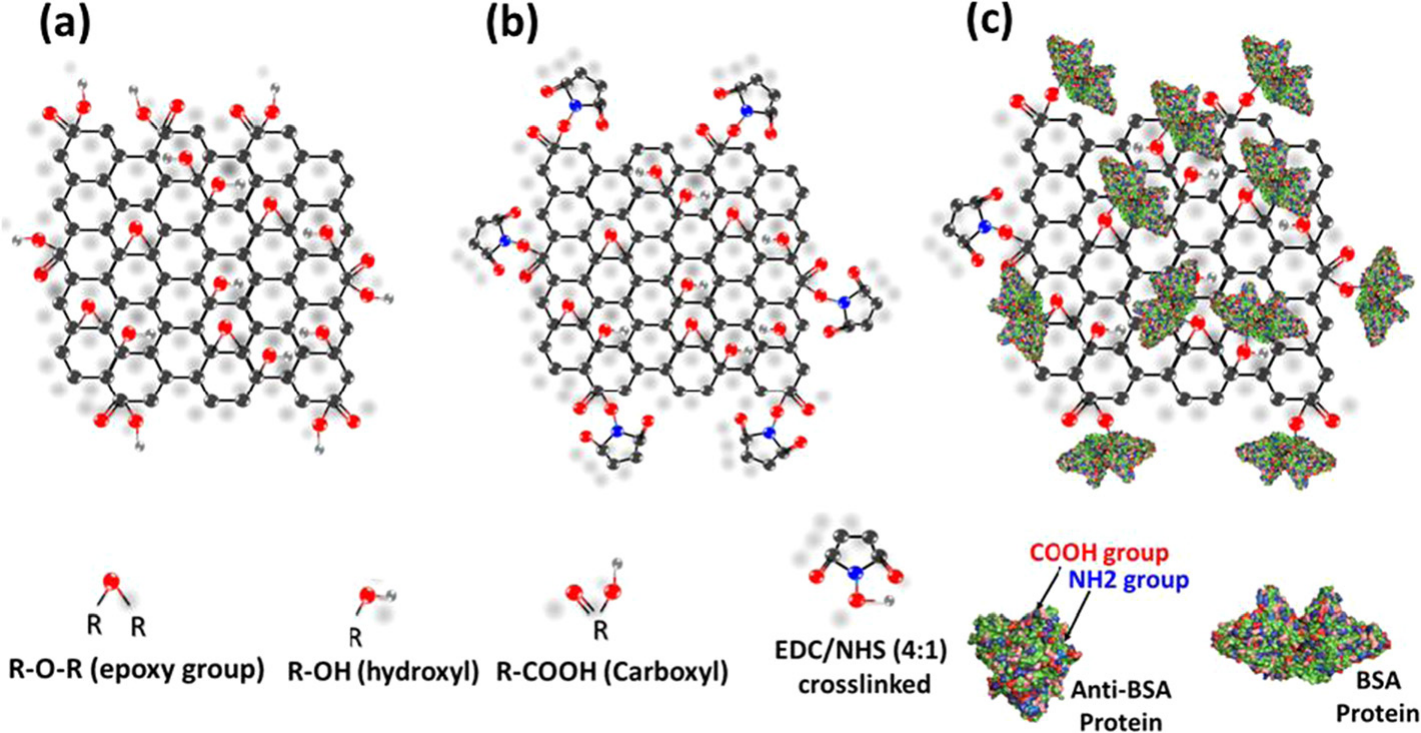
**GOS, terminal groups, and carboxyl groups. (a)** Molecular structure of GOS. **(b)** Modification of terminal groups (-COOH) of monolayers of GOS film by surface-confined ester reactions. **(c)** Carboxyl groups ending in -COOH cause GOS surface to exhibit affinity for NH2 end of protein.

The GOS (Graphene Laboratories Inc., Calverton, NY, USA) was manufactured by Hummer’s method and diluted in water to a concentration of 2 mg/ml. In general, the oxide of a graphene material contains an epoxy group, a hydroxyl group, and a carboxyl group. Therefore, more efficient chemical modification methods and means of activating the carboxyl groups on the GOS surface are sought. The GOS immobilization was chemically modified by a reaction with a 4:1 ratio of *N*-hydroxysulfosuccinimide (NHS)/*N*-ethyl-3-(3-dimethylaminopropyl) carbodiimide (EDC). Carboxylic acid groups of GOS were converted to reactive NHS esters using EDC and NHS, and GOS were subsequently immobilized by reacting its NHS-activated carboxylic acid groups. This method can convert carboxyl groups to amine-reactive NHS esters that immobilize hydrocarbon chains, as shown in Figure 2b. The activated surfaces of the GOS reacted with the amine groups of the BSA protein, subsequently forming a strongly covalent bond, as shown in Figure 2c. Analytical results suggest that in addition to improving the protein compatibility of this GOS material, GOS immobilization to EDC/NHS-crosslinks can be used to prepare a chemically modified GOS film-based SPR chip specifically for analysis in a protein sample solution [10,36,37].

### Kinetic analysis of bimolecular interactions at surface

SPR sensorgrams include real-time information on the changes in mass that are caused by binding in a bimolecular interaction, such as that between probe [P] and target [T], as follows [38,39].

(1) $$\left[P\right]+{\left[T\right]}_{\underset{\mathrm{Kd}}{\leftarrow }}^{\overset{\mathrm{Ka}}{\to }}\;\left[\mathrm{PT}\right]$$

In a bimolecular competition experiment with a probe for the target that is present both on the sensor surface and in solution, the complex [PT] is formed, and under the two binding equilibria, the dissociation constant *K*_A_ and dissociation constant *K*_D_ are given by Equation 2.

(2) $$K_{A}=\frac{\left[\mathrm{PT}\right]}{\left[P\right]\left[T\right]}=\frac{k_{a}}{k_{d}},\;\mathrm{with}\;K_{D}=\frac{1}{K_{A}}=\frac{k_{d}}{k_{a}}$$

where *k*_a_ and *k*_d_ are the association and dissociation rate constants for the formation and dissociation of the complex [PT].

**Figure 3:**
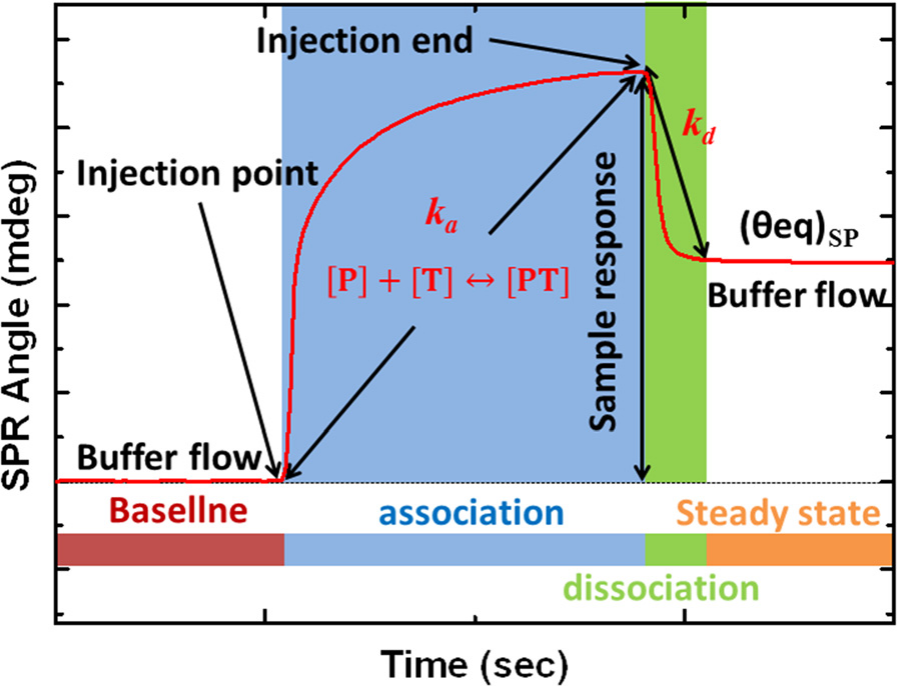
Time course for value of SPR sensorgrams in analysis of interaction that involves bimolecular association and dissociation.

Figure 3 shows an analysis of the cyclic sensorgram of the change in the refractive index of the liquid phase close to the sensor chip surface in the SPR experiments. The amount of complex [PT] is proportional to the shift in SPR angle (mdeg). The observed shift in the SPR angle is the sum of the shift that is caused by the association of [T] with [P] and that is caused by the difference between the refractive indices of the solution and the baseline buffer. The shift in the SPR angle is recorded as a function of time in the sensorgram. At equilibrium, the fraction of the surface that is covered reaches a steady state and this equilibrium surface coverage (*θ*_eq_)_SP_ is given by the Langmuir absorption isotherm, [40]

(3) $${\left(\theta_{\mathrm{eq}}\right)}_{\mathrm{SP}}=\frac{K_{\mathrm{ads}}\left[T\right]}{1+K_{\mathrm{ads}}\left[T\right]}$$

where the Langmuir absorption coefficient (*K*_abs_) is defined as *K*_abs_ *= k*_a_*/k*_d_.

Based on Fresnel’s equations, given the reflection coefficient, the SP wave vectors for the Au-GOS-BSA boundary, and the coupler matching condition of the SPR are as given by Equation 4.

(4) $$K_{x}=k_{0}\sqrt{\varepsilon_{p}}\text{sin}\left(\theta_{\mathrm{eq}}\right)=k_{0}\sqrt{\frac{\varepsilon_{d}\varepsilon_{m}}{\varepsilon_{d}+\varepsilon_{m}}}=K_{\mathrm{SP}}$$

where *K*_*x*_ is the wave-vector parallel to the surface form which light is reflected, *K*_0_ is the wave-vector in a vacuum, and *K*_sp_ is the SP wave-vector that is parallel to the interfaces between the metal and the dielectric. *θ*_eq_ is the SPR angle at equilibrium, *ε*_p_ is the refractive index of the prism, and *ε*_m_ and *ε*_d_ are the metal and dielectric constants of the sample, respectively.

## Results and discussion

### Analysis of sensitivity of interaction between GOS and BSA

**Figure 4:**
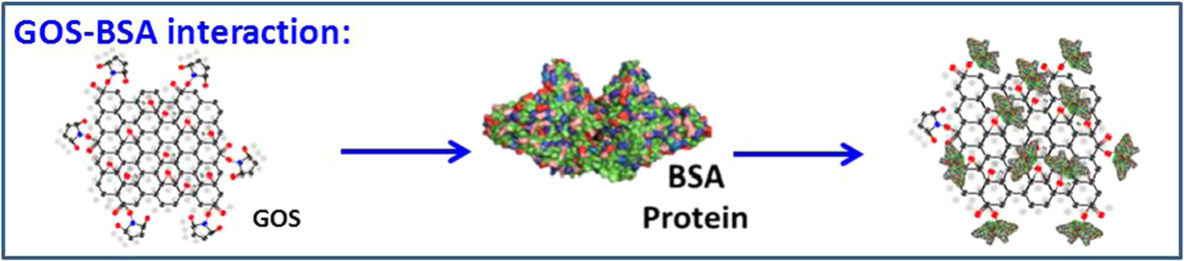
**GOS-BSA interaction.** GOS is immobilized on a planar immobilization film, which is a few tens of nanometers thick, and is readily accessible by analytic BSA protein with which it undergoes specific interactions.

Two-dimensional GOS surfaces can detect a large area, in which the evanescent field decays exponentially with the distance beyond 600 nm from the metal. Figure 4 shows the interaction of a GOS with BSA. GOS performs a spacing function BSA and GOS, which increases the accessibility of the immobilized GOS.

### Kinetic analysis of interaction between GOS and BSA

**Figure 5:**
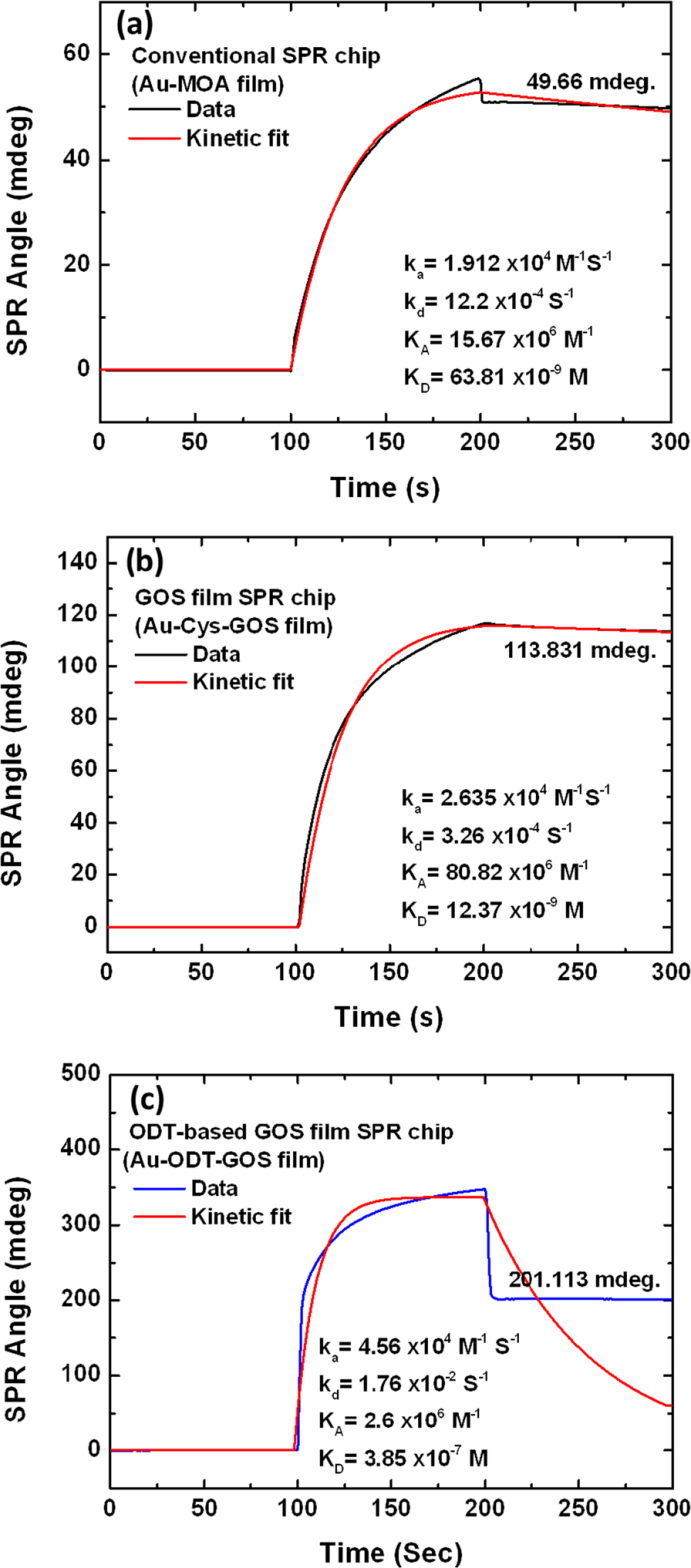
**SPR sensorgrams obtained in response to BSA, at concentration of 100 μg/ml, flowing over surfaces of films. (a)** Interaction with conventional SPR chip based on Au-MOA film, **(b)** interaction with the Cys-based GOS film-based SPR chip, and **(c)** interaction with ODT-based GOS film-based SPR chip. Association rate (*k*_a_), dissociation rate (*k*_d_), affinity constant (*K*_A_), and dissociation constant (*K*_D_) were obtained from fitted curves.

Molecular kinetics of the interactions of the three sensor films and the protein are analyzed. Figure 5 presents the SPR sensorgrams (BI-3000G SPR system) of a Au-MOA film (conventional SPR chip) (Figure 5a), a Au-Cys-GOS film (GOS film-based SPR chip) (Figure 5b), and a Au-ODT-GOS film (ODT-based GOS film-based SPR chip) (Figure 5c), in response to solutions of BSA with a concentration of 100 μg/ml in phosphate buffered saline (PBS) buffer. The affinity constants (*K*_A_) of 100 μg/ml BSA on the ODT-based GOS film-based SPR chip, the conventional SPR chip, and the GOS film-based SPR chip were 2.6 × 10^6^ M^-1^, 15.67 × 10^6^ M^-1^, and 80.82 × 10^6^ M^-1^, respectively. The ratio of the affinities of the ODT-based GOS film-based SPR chip, conventional chip, and GOS film SPR chip was 1:6:31 times. The results demonstrate that this Cys-modified Au surface excellently immobilized a GOS film in an SPR chip.

**Figure 6:**
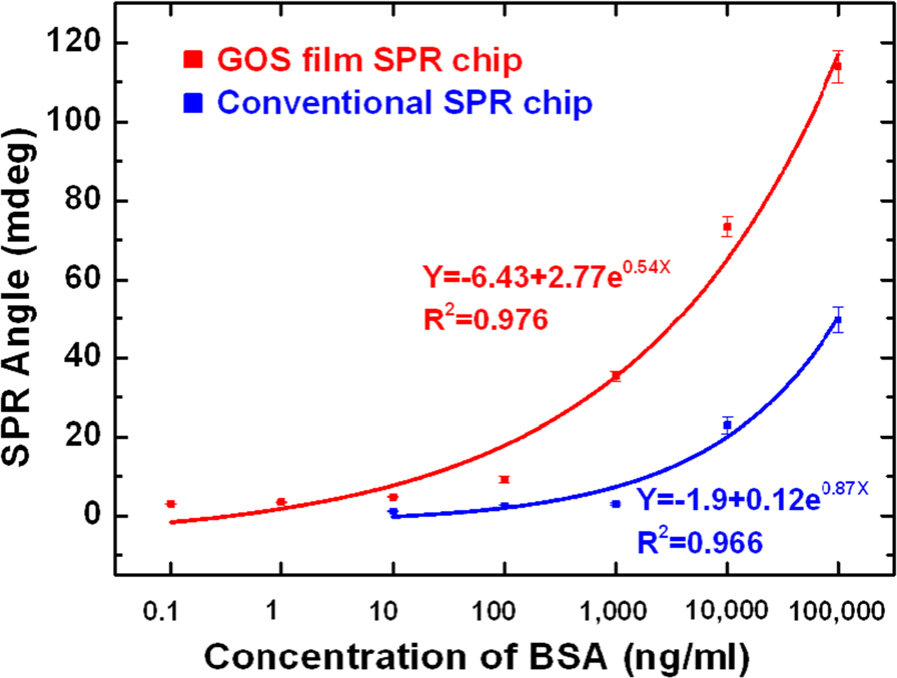
**Response of sensor film to various concentrations of BSA.** Calibration curves for detection of BSA by GOS film-based SPR chip and conventional SPR chip.

Figure 6 shows SPR response curves of conventional SPR chip and GOS film-based SPR chip, which exhibits higher sensitivity. In the detection of BSA protein, the limit of detection (LOD) of the conventional SPR chip was 10 ng/ml; that of the GOS film-based SPR chip was as low as 100 pg/ml. This GOS film-based SPR chip had a limit of detection (LOD) for BSA that was 1/100 that of the conventional Au-film-based sensor. These results were consistent with the calibration curves. The calibration curves were fitted by *y* = -6.43 + 2.77 e^0.54*x*^ (correlation coefficient, *R*^2^ = 0.976) for the GOS film-based SPR chip, and *y* = -1.9 + 0.12 e^0.87*x*^ (correlation coefficient, *R*^2^ = 0.966) for the conventional SPR chip, where *x* is the concentration of BSA and *y* is the SPR angle (*θ*).

### Biomolecular interaction analysis using BSA and anti-BSA

**Figure 7:**
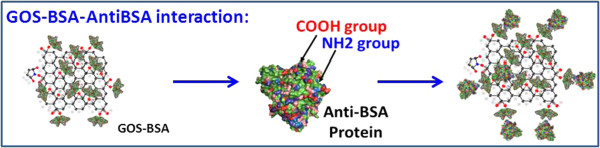
**GOS-BSA-anti-BSA interaction.** GOS-BSA is immobilized on a planar immobilization film that is a few hundreds of nanometers thick and is readily accessible to analytic anti-BSA protein with which it undergoes particular interactions.

**Figure 8:**
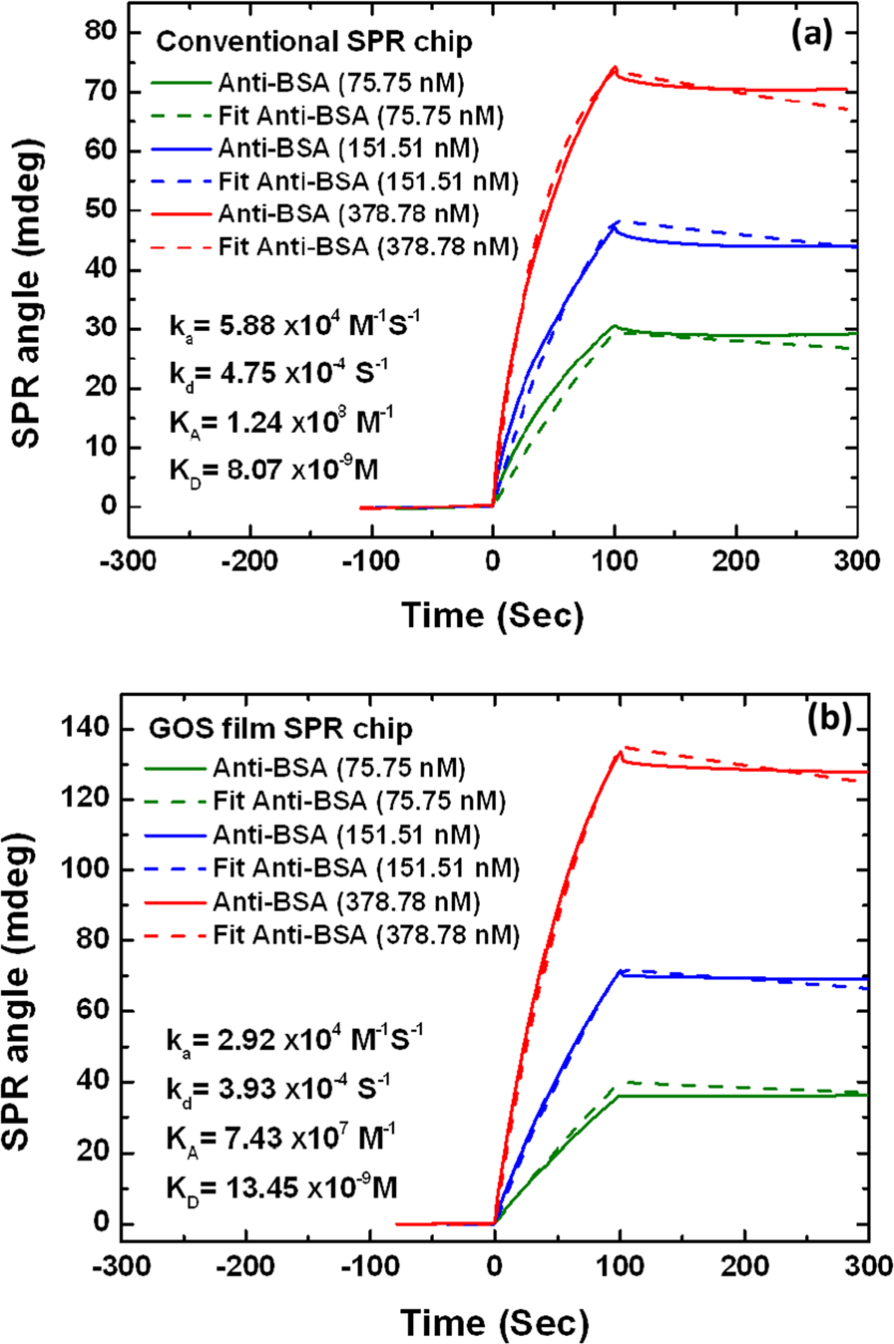
**Sensorgram of immobilization of BSA 100 μg/ml on sensor chip in real time.** Various detected concentrations of anti-BSA on **(a)** conventional SPR chip and **(b)** GOS film-based SPR chip.

To evaluate the sensitivity and specificity of the developed immunoassay film in the on-site detection of anti-bovine serum albumin (Anti-BSA; Sigma, Chemical Company, St. Louis, MO, USA), an anti-BSA antibody sample was diluted to 378.78, 151.51, and 75.75 nM by adding PBS buffer. Figure 7 schematically depicts the Au-Cys-GOS-BSA-enhanced SPR sensor for anti-BSA.Figure 8 plots the SPR response in the adsorption of anti-BSA proteins on the GOS film-based SPR chip. Real-time SPR angle signals are obtained for 75.75, 151.51, and 378.78 nM anti-BSA antibodies on the conventional SPR film chip at 26.1759, 39.4802, and 63.8839 mdeg (millimeter degree), as shown in Figure 8a. Real-time SPR angle signals are obtained for 75.75, 151.51, and 378.78 nM anti-BSA proteins on the immunoassay film chip at 36.1867, 69.1671, and 127.7401 mdeg, as shown in Figure 8b.

**Figure 9:**
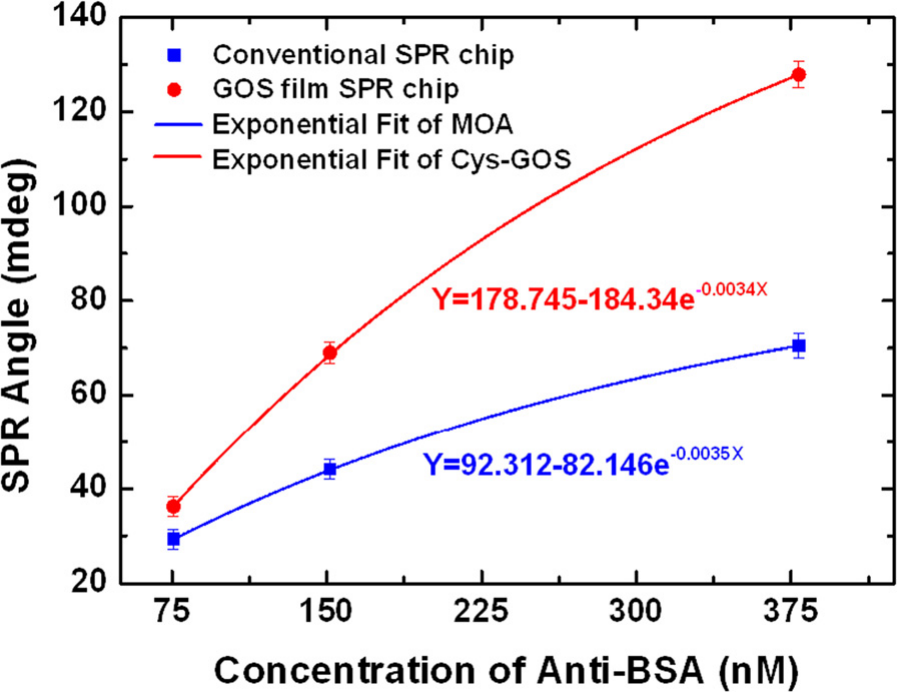
Equilibrium analysis of binding of anti-BSA protein to a high-affinity BSA protein.

Binding affinity was determined using anti-BSA protein concentrations of 75 to 378.78 nM. Since the immunoassay analyses were carried out using the same protein, BSA, with the anti-BSA interaction, the results are similar to those of the kinetic analysis, as shown in Figure 8a,b. The responses were measured against the concentration for the protein-protein interactions. Comparing the immunoassay sensitivity of the GOS film-based SPR chip and the conventional SPR chip reveals that the former exhibits an SPR angle shift that is 1.4 times higher than that of the latter at the low concentration of 75.75 nM and twice that of the latter at the high concentration of 378.78 nM, as shown in Figure 9. The anti-BSA concentration was exponentially fitted in the range of 75.75 to 378.78 nM. Additionally, the exponential regression equations of the slope of each fitted curve were as follows: 178.745 to 184.34 e^-0.034*x*^ for the GOS film-based SPR chip and 92.312 to 82.146 e^-0.0035*x*^ for the conventional SPR chip.

## Conclusions

In summary, a GOS film was developed for binding with proteins based on SPR analysis for the purpose of immunoassay sensing. The GOS film-based SPR chip herein had a BSA concentration detection limit of as low as 100 pg/ml, which was 1/100th that of the conventional SPR chip. Additionally, in immunoassay detection, the GOS film-based SPR chip was highly sensitive at a low concentration of 75.75 nM, exhibiting an SPR angle shift of 1.4 times that of the conventional chip, and exhibited an SPR angle shift of two times that of the conventional chip at a high concentration of 378.78 nM. Finally, we believe that the fact that the GOS can be chemically modified to increase its SPR sensitivity can be exploited in clinical diagnostic protein-protein interaction applications, especially in cases in which tumor molecular detection is feasible.

## Competing interests

The authors declare that they have no competing interests.

## Authors’ contributions

N-FC participated in the design of the study and performed the statistical analysis and drafted the manuscript. T-YH carried out the immunoassays and performed the statistical analysis. H-CL and K-CL conceived the study and participated in its design and coordination. All authors read and approved the final manuscript.
